# 3,4-Dimeth­oxy-*N*-((*E*)-4-{[1-(prop-2-en-1-yl)-1*H*-1,2,3-triazol-4-yl]meth­oxy}benzyl­idene)aniline

**DOI:** 10.1107/S1600536812026761

**Published:** 2012-06-16

**Authors:** Mehmet Akkurt, Aliasghar Jarrahpour, Hashem Sharghi, Mehdi Mohammadi Chermahini, Pezhman Shiri, Orhan Büyükgüngör

**Affiliations:** a Department of Physics, Faculty of Sciences, Erciyes University, 38039 Kayseri, Turkey; b Department of Chemistry, College of Sciences, Shiraz University, 71454 Shiraz, Iran; c Department of Physics, Faculty of Arts and Sciences, Ondokuz Mayıs University, 55139 Samsun, Turkey

## Abstract

In the title compound, C_21_H_22_N_4_O_3_, the triazole ring is planar [maximum deviaton = 0.004 (1) Å] and makes dihedral angles of 26.21 (8) and 38.66 (8)° with the two benzene rings. In the crystal, mol­ecules are linked by C—H⋯O hydrogen bonds, forming zigzag chains along [1-11]. In addition, a weak C—H⋯π intreraction is also observed.

## Related literature

For background on the importance of triazole derivatives and their uses, see: da Silva *et al.* (2011 ▶); Hranjec *et al.* (2011 ▶); Shi & Zhou (2011 ▶); Yap & Weinreb (2006 ▶); Yu *et al.* (2006 ▶).
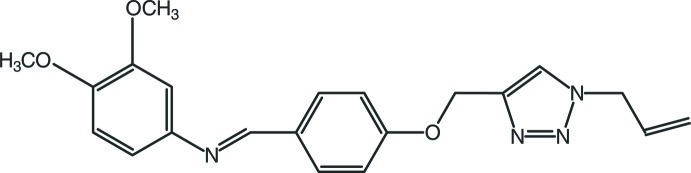


## Experimental

### 

#### Crystal data

C_21_H_22_N_4_O_3_
*M* *_r_* = 378.43
Triclinic, 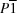

*a* = 5.8144 (4) Å
*b* = 11.9677 (8) Å
*c* = 14.1019 (10) Å
α = 85.803 (6)°
β = 80.497 (6)°
γ = 80.434 (5)°
*V* = 953.30 (12) Å^3^
*Z* = 2
Mo *K*α radiation
μ = 0.09 mm^−1^
*T* = 296 K
0.73 × 0.41 × 0.21 mm

#### Data collection

Stoe IPDS 2 diffractometer
Absorption correction: integration (*X-RED32*; Stoe & Cie, 2002 ▶) *T*_min_ = 0.957, *T*_max_ = 0.981
15258 measured reflections
3919 independent reflections
2795 reflections with *I* > 2σ(*I*)
*R*_int_ = 0.050

#### Refinement

*R*[*F* ^2^ > 2σ(*F* ^2^)] = 0.041
*wR*(*F* ^2^) = 0.107
*S* = 1.04
3919 reflections
253 parameters
H-atom parameters constrained
Δρ_max_ = 0.14 e Å^−3^
Δρ_min_ = −0.18 e Å^−3^

### 

Data collection: *X-AREA* (Stoe & Cie, 2002 ▶); cell refinement: *X-AREA*; data reduction: *X-RED32* (Stoe & Cie, 2002 ▶); program(s) used to solve structure: *SHELXS97* (Sheldrick, 2008 ▶); program(s) used to refine structure: *SHELXL97* (Sheldrick, 2008 ▶); molecular graphics: *ORTEP-3* (Farrugia, 1997 ▶); software used to prepare material for publication: *WinGX* (Farrugia, 1999 ▶).

## Supplementary Material

Crystal structure: contains datablock(s) global, I. DOI: 10.1107/S1600536812026761/gk2500sup1.cif

Structure factors: contains datablock(s) I. DOI: 10.1107/S1600536812026761/gk2500Isup2.hkl

Supplementary material file. DOI: 10.1107/S1600536812026761/gk2500Isup3.cml

Additional supplementary materials:  crystallographic information; 3D view; checkCIF report

## Figures and Tables

**Table 1:** Hydrogen-bond geometry (Å, °) *Cg*2 is the centroid of the C1–C6 benzene ring.

*D*—H⋯*A*	*D*—H	H⋯*A*	*D*⋯*A*	*D*—H⋯*A*
C19—H19*A*⋯O2^i^	0.97	2.54	3.385 (2)	146
C16—H16*A*⋯*Cg*2^ii^	0.97	2.87	3.6647 (18)	140

Symmetry codes: (i) 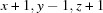; (ii) 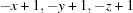.

**Fig. 1.**
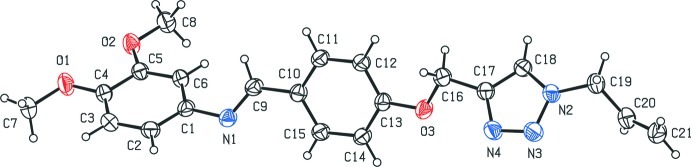
The title molecule shown with the displacement ellipsoids for non-H atoms at the 30% probability level.

**Fig. 2.**
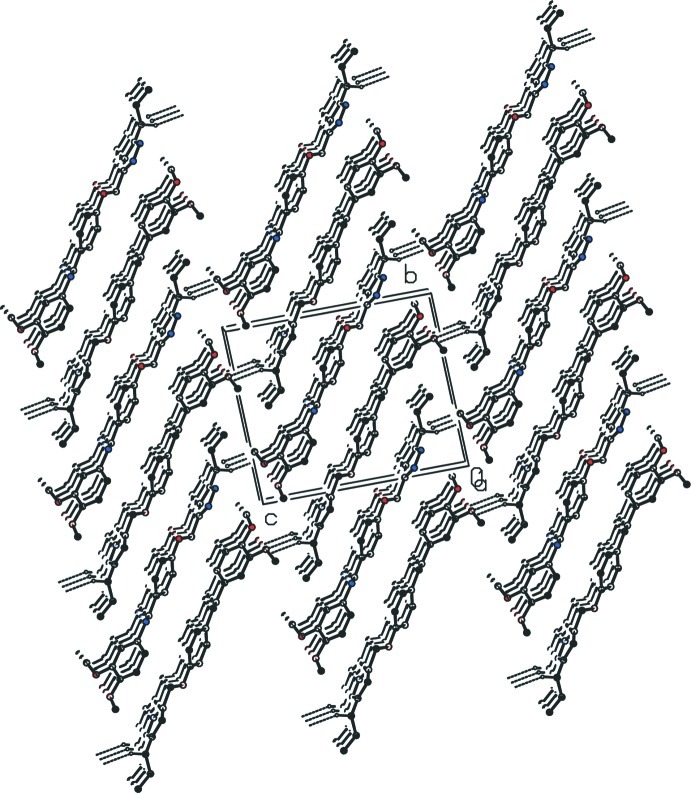
Crystal packing and hydrogen bonding diagram of the title compound shown down the a axis. H atoms not participating in hydrogen bonding have been omitted for clarity.

## supplementary crystallographic information

### Comment

Schiff bases are aldehyde- or ketone-like compounds in which the carbonyl group is replaced by an imine or azomethine group. They are widely used for industrial purposes and also exhibit a broad range of biological activities (da Silva *et al.*, 2011). These compounds represent an important class of organic compounds, especially in the medicinal and pharmaceutical field. Thus, development and synthesis of novel Schiff base derivatives as potential chemotherapeutics still attracts attention of organic and medicinal chemists (Hranjec *et al.*, 2011). 1,2,3-Triazoles are nitrogen heterocycles, which have a number of important industrial, agrochemical, and pharmaceutical uses (Yap & Weinreb, 2006). Triazole derivatives also display a broad range of biological activity, showing potential applications as antitumor, antibacterial, antifungal and antiviral agents (Yu *et al.*, 2006). Recently, some triazole derivatives have been reported to exhibit good anti-MRSA potency (Shi & Zhou, 2011). Therefore, compound (I), which has the two mentioned features, was synthesized and its X-ray studies is reported here.

The title compound, (Fig. 1), has a non-planar conformation. The N2–N4/C17—C18 triazole ring is essentially planar with a maximum deviaton of 0.004 (1) Å. The dihedral angles between the rings in (I) are: A/B = 26.21 (8), A/C = 38.66 (8) and B/C = 64.87 (7)°, where A, B and C define the C1–C6 and C10–C15 benzene rings, and the N2–N4/C17—C18 triazole ring, respectively.

The crystal packing is stabilized by intermolecular C—H···O hydrogen bonding interactions (Table 1) forming zigzag chains along the [1 -1 1] direction (Fig. 2). Furthermore, a weak intermolecular C—H···π interaction contributes to the stability of the crystal structure.

### Experimental

Reaction of 4-((1-allyl-1*H*-1, 2, 3-triazol-4-yl)methoxy)benzaldehyde with 3,4-dimethoxyaniline in refluxing ethanol gave the title compound. Recrystallization from ethanol gave brown crystals in 85% yield. [mp: 363–365 K]. IR (KBr, cm^-1^): 1573 (N=N), 1612 (C═N). ^1^H-NMR (250 MHz, CDCl_3_) δ (p.p.m): 3.87–3.90 (2OMe, s, 6H), 4.97 (d, 2H, J=6.0 Hz), 5.25–5.37 (m, 4H), 5.93–6.1(m, 1H), 6.84–7.89(m, ArH, 7H), 7.62(s, 1H, Htriazole), 8.41(HC═N, s, 1H). 13 C-NMR (CDCl_3_) δ (p.p.m):62.8 (N—CH_2_), 55.9–56.0 (2OMe), 62.0 (O—CH_2_), 105.7–157.9 (C=C aromatic carbons and triazole), 160.96(C=N).

### Refinement

H atoms were positioned geometrically with C—H = 0.93, 0.96 and 0.97 Å, and refined using a riding model with *U*_iso_(H) = 1.2 or 1.5*U*_eq_(C).

### Crystal data

**Table:**

C_21_H_22_N_4_O_3_	*Z* = 2
*M**_r_* = 378.43	*F*(000) = 400
Triclinic, *P*1	*D*_x_ = 1.318 Mg m^−^^3^
Hall symbol: -P 1	Mo *K*α radiation, λ = 0.71073 Å
*a* = 5.8144 (4) Å	Cell parameters from 16791 reflections
*b* = 11.9677 (8) Å	θ = 2.2–27.3°
*c* = 14.1019 (10) Å	µ = 0.09 mm^−^^1^
α = 85.803 (6)°	*T* = 296 K
β = 80.497 (6)°	Prism, brown
γ = 80.434 (5)°	0.73 × 0.41 × 0.21 mm
*V* = 953.30 (12) Å^3^	

### Data collection

**Table:**

Stoe IPDS 2 diffractometer	3919 independent reflections
Radiation source: sealed X-ray tube, 12 x 0.4 mm long-fine focus	2795 reflections with *I* > 2σ(*I*)
Plane graphite monochromator	*R*_int_ = 0.050
Detector resolution: 6.67 pixels mm^-1^	θ_max_ = 26.5°, θ_min_ = 2.2°
ω scans	*h* = −7→7
Absorption correction: integration (*X-RED32*; Stoe & Cie, 2002)	*k* = −15→15
*T*_min_ = 0.957, *T*_max_ = 0.981	*l* = −17→17
15258 measured reflections	

### Refinement

**Table:**

Refinement on *F*^2^	Primary atom site location: structure-invariant direct methods
Least-squares matrix: full	Secondary atom site location: difference Fourier map
*R*[*F*^2^ > 2σ(*F*^2^)] = 0.041	Hydrogen site location: inferred from neighbouring sites
*wR*(*F*^2^) = 0.107	H-atom parameters constrained
*S* = 1.04	*w* = 1/[σ^2^(*F*_o_^2^) + (0.0603*P*)^2^] where *P* = (*F*_o_^2^ + 2*F*_c_^2^)/3
3919 reflections	(Δ/σ)_max_ < 0.001
253 parameters	Δρ_max_ = 0.14 e Å^−^^3^
0 restraints	Δρ_min_ = −0.18 e Å^−^^3^

### Special details

**Table:**

Geometry. Bond distances, angles *etc*. have been calculated using the rounded fractional coordinates. All su's are estimated from the variances of the (full) variance-covariance matrix. The cell e.s.d.'s are taken into account in the estimation of distances, angles and torsion angles
Refinement. Refinement on *F*^2^ for ALL reflections except those flagged by the user for potential systematic errors. Weighted *R*-factors *wR* and all goodnesses of fit *S* are based on *F*^2^, conventional *R*-factors *R* are based on *F*, with *F* set to zero for negative *F*^2^. The observed criterion of *F*^2^ > σ(*F*^2^) is used only for calculating -*R*-factor-obs *etc*. and is not relevant to the choice of reflections for refinement. *R*-factors based on *F*^2^ are statistically about twice as large as those based on *F*, and *R*-factors based on ALL data will be even larger.

### Fractional atomic coordinates and isotropic or equivalent isotropic displacement parameters (Å^2^)

**Table:**

	*x*	*y*	*z*	*U*_iso_*/*U*_eq_	
O1	−0.04640 (19)	0.88296 (10)	0.07531 (10)	0.0725 (4)	
O2	0.35022 (17)	0.75647 (9)	0.03759 (8)	0.0615 (4)	
O3	0.67186 (18)	0.09568 (8)	0.56736 (8)	0.0618 (4)	
N1	0.1657 (2)	0.50851 (10)	0.32214 (9)	0.0553 (4)	
N2	1.0236 (2)	−0.14989 (10)	0.76913 (9)	0.0557 (4)	
N3	0.7884 (2)	−0.14280 (12)	0.78848 (11)	0.0693 (5)	
N4	0.6981 (2)	−0.05436 (12)	0.73864 (11)	0.0661 (5)	
C1	0.1137 (2)	0.60697 (11)	0.26194 (11)	0.0503 (4)	
C2	−0.1013 (2)	0.67614 (12)	0.28129 (11)	0.0561 (5)	
C3	−0.1602 (3)	0.77029 (13)	0.22197 (12)	0.0581 (5)	
C4	−0.0060 (2)	0.79558 (11)	0.14080 (11)	0.0526 (5)	
C5	0.2124 (2)	0.72537 (11)	0.12021 (10)	0.0495 (4)	
C6	0.2718 (2)	0.63275 (11)	0.17977 (11)	0.0508 (4)	
C7	−0.2711 (3)	0.95187 (13)	0.08467 (16)	0.0732 (6)	
C8	0.5684 (3)	0.68677 (14)	0.00812 (13)	0.0653 (6)	
C9	0.3712 (3)	0.48356 (12)	0.34398 (11)	0.0537 (5)	
C10	0.4505 (2)	0.38187 (12)	0.40066 (11)	0.0504 (4)	
C11	0.6468 (2)	0.37782 (12)	0.44608 (12)	0.0554 (5)	
C12	0.7232 (2)	0.28590 (12)	0.50323 (11)	0.0550 (5)	
C13	0.6079 (2)	0.19201 (11)	0.51366 (11)	0.0497 (4)	
C14	0.4171 (2)	0.19244 (12)	0.46571 (11)	0.0539 (5)	
C15	0.3388 (2)	0.28612 (12)	0.41111 (11)	0.0534 (5)	
C16	0.8385 (3)	0.10194 (13)	0.62971 (13)	0.0608 (5)	
C17	0.8768 (2)	−0.00575 (12)	0.68750 (11)	0.0523 (4)	
C18	1.0838 (2)	−0.06698 (12)	0.70649 (11)	0.0545 (5)	
C19	1.1770 (3)	−0.23609 (13)	0.81922 (14)	0.0659 (6)	
C20	1.1179 (3)	−0.35129 (13)	0.81555 (13)	0.0613 (5)	
C21	1.1076 (3)	−0.42391 (14)	0.88837 (14)	0.0708 (6)	
H2	−0.20840	0.65930	0.33500	0.0670*	
H3	−0.30470	0.81690	0.23690	0.0700*	
H6	0.41750	0.58680	0.16560	0.0610*	
H7A	−0.30230	0.98770	0.14520	0.0880*	
H7B	−0.38990	0.90580	0.08210	0.0880*	
H7C	−0.27330	1.00890	0.03310	0.0880*	
H8A	0.54070	0.61200	−0.00200	0.0780*	
H8B	0.66660	0.68280	0.05710	0.0780*	
H8C	0.64580	0.71830	−0.05080	0.0780*	
H9	0.47820	0.53310	0.32260	0.0640*	
H11	0.72860	0.43920	0.43750	0.0660*	
H12	0.85140	0.28650	0.53480	0.0660*	
H14	0.34230	0.12890	0.47070	0.0650*	
H15	0.20890	0.28590	0.38050	0.0640*	
H16A	0.78020	0.16410	0.67210	0.0730*	
H16B	0.98690	0.11620	0.59190	0.0730*	
H18	1.23550	−0.05380	0.68110	0.0650*	
H19A	1.16170	−0.21710	0.88590	0.0790*	
H19B	1.34010	−0.23560	0.79000	0.0790*	
H20	1.08630	−0.37280	0.75750	0.0740*	
H21A	1.13830	−0.40490	0.94730	0.0850*	
H21B	1.06940	−0.49510	0.88170	0.0850*	

### Atomic displacement parameters (Å^2^)

**Table:**

	*U*^11^	*U*^22^	*U*^33^	*U*^12^	*U*^13^	*U*^23^
O1	0.0606 (6)	0.0700 (7)	0.0781 (8)	0.0077 (5)	−0.0144 (6)	0.0221 (6)
O2	0.0562 (6)	0.0660 (6)	0.0546 (7)	0.0023 (5)	−0.0065 (5)	0.0151 (5)
O3	0.0753 (7)	0.0498 (5)	0.0673 (7)	−0.0155 (5)	−0.0325 (6)	0.0124 (5)
N1	0.0600 (7)	0.0532 (6)	0.0553 (8)	−0.0148 (5)	−0.0159 (6)	0.0100 (6)
N2	0.0519 (7)	0.0592 (7)	0.0563 (8)	−0.0087 (5)	−0.0153 (6)	0.0104 (6)
N3	0.0569 (8)	0.0777 (9)	0.0704 (9)	−0.0128 (6)	−0.0112 (7)	0.0244 (7)
N4	0.0511 (7)	0.0728 (8)	0.0710 (9)	−0.0081 (6)	−0.0111 (6)	0.0185 (7)
C1	0.0548 (8)	0.0477 (7)	0.0528 (8)	−0.0149 (6)	−0.0180 (6)	0.0053 (6)
C2	0.0505 (8)	0.0608 (8)	0.0587 (9)	−0.0154 (7)	−0.0097 (7)	0.0033 (7)
C3	0.0459 (7)	0.0590 (8)	0.0685 (10)	−0.0034 (6)	−0.0123 (7)	−0.0003 (7)
C4	0.0514 (8)	0.0505 (7)	0.0571 (9)	−0.0059 (6)	−0.0183 (6)	0.0066 (7)
C5	0.0496 (7)	0.0528 (7)	0.0479 (8)	−0.0092 (6)	−0.0148 (6)	0.0040 (6)
C6	0.0493 (7)	0.0500 (7)	0.0538 (9)	−0.0045 (6)	−0.0158 (6)	0.0039 (6)
C7	0.0631 (10)	0.0586 (9)	0.0952 (14)	0.0050 (7)	−0.0242 (9)	0.0085 (9)
C8	0.0573 (9)	0.0723 (10)	0.0603 (10)	−0.0005 (7)	−0.0056 (7)	0.0072 (8)
C9	0.0585 (8)	0.0535 (8)	0.0524 (9)	−0.0174 (6)	−0.0138 (7)	0.0070 (6)
C10	0.0540 (8)	0.0515 (7)	0.0457 (8)	−0.0107 (6)	−0.0083 (6)	0.0052 (6)
C11	0.0584 (8)	0.0516 (7)	0.0599 (9)	−0.0183 (6)	−0.0156 (7)	0.0094 (7)
C12	0.0539 (8)	0.0539 (8)	0.0611 (9)	−0.0143 (6)	−0.0191 (7)	0.0072 (7)
C13	0.0539 (8)	0.0476 (7)	0.0478 (8)	−0.0083 (6)	−0.0112 (6)	0.0054 (6)
C14	0.0597 (8)	0.0498 (7)	0.0565 (9)	−0.0184 (6)	−0.0154 (7)	0.0062 (7)
C15	0.0536 (8)	0.0573 (8)	0.0529 (9)	−0.0147 (6)	−0.0167 (6)	0.0060 (7)
C16	0.0618 (9)	0.0590 (8)	0.0662 (10)	−0.0142 (7)	−0.0253 (7)	0.0123 (7)
C17	0.0522 (8)	0.0542 (7)	0.0512 (8)	−0.0095 (6)	−0.0124 (6)	0.0046 (6)
C18	0.0496 (8)	0.0582 (8)	0.0554 (9)	−0.0111 (6)	−0.0094 (6)	0.0083 (7)
C19	0.0655 (9)	0.0647 (9)	0.0701 (11)	−0.0091 (7)	−0.0285 (8)	0.0174 (8)
C20	0.0631 (9)	0.0614 (9)	0.0583 (10)	0.0010 (7)	−0.0173 (7)	−0.0014 (8)
C21	0.0779 (11)	0.0622 (9)	0.0745 (12)	−0.0105 (8)	−0.0244 (9)	0.0093 (9)

### Geometric parameters (Å, º)

**Table:**

O1—C4	1.3603 (19)	C16—C17	1.478 (2)
O1—C7	1.416 (2)	C17—C18	1.3572 (18)
O2—C5	1.3658 (17)	C19—C20	1.482 (2)
O2—C8	1.415 (2)	C20—C21	1.296 (3)
O3—C13	1.3651 (17)	C2—H2	0.9300
O3—C16	1.426 (2)	C3—H3	0.9300
N1—C1	1.4215 (18)	C6—H6	0.9300
N1—C9	1.265 (2)	C7—H7A	0.9600
N2—N3	1.3392 (17)	C7—H7B	0.9600
N2—C18	1.3313 (19)	C7—H7C	0.9600
N2—C19	1.468 (2)	C8—H8A	0.9600
N3—N4	1.311 (2)	C8—H8B	0.9600
N4—C17	1.3528 (19)	C8—H8C	0.9600
C1—C2	1.3780 (18)	C9—H9	0.9300
C1—C6	1.405 (2)	C11—H11	0.9300
C2—C3	1.384 (2)	C12—H12	0.9300
C3—C4	1.381 (2)	C14—H14	0.9300
C4—C5	1.4008 (18)	C15—H15	0.9300
C5—C6	1.3732 (19)	C16—H16A	0.9700
C9—C10	1.458 (2)	C16—H16B	0.9700
C10—C11	1.3906 (18)	C18—H18	0.9300
C10—C15	1.3956 (19)	C19—H19A	0.9700
C11—C12	1.372 (2)	C19—H19B	0.9700
C12—C13	1.3883 (19)	C20—H20	0.9300
C13—C14	1.3904 (18)	C21—H21A	0.9300
C14—C15	1.370 (2)	C21—H21B	0.9300
			
C4—O1—C7	119.09 (13)	C4—C3—H3	120.00
C5—O2—C8	118.37 (12)	C1—C6—H6	120.00
C13—O3—C16	116.35 (11)	C5—C6—H6	120.00
C1—N1—C9	118.68 (12)	O1—C7—H7A	109.00
N3—N2—C18	110.57 (12)	O1—C7—H7B	109.00
N3—N2—C19	120.68 (13)	O1—C7—H7C	109.00
C18—N2—C19	128.62 (13)	H7A—C7—H7B	109.00
N2—N3—N4	107.23 (12)	H7A—C7—H7C	109.00
N3—N4—C17	108.59 (12)	H7B—C7—H7C	110.00
N1—C1—C2	119.65 (13)	O2—C8—H8A	109.00
N1—C1—C6	121.52 (11)	O2—C8—H8B	109.00
C2—C1—C6	118.75 (13)	O2—C8—H8C	109.00
C1—C2—C3	120.94 (14)	H8A—C8—H8B	110.00
C2—C3—C4	120.51 (14)	H8A—C8—H8C	109.00
O1—C4—C3	126.10 (13)	H8B—C8—H8C	109.00
O1—C4—C5	114.93 (13)	N1—C9—H9	118.00
C3—C4—C5	118.95 (13)	C10—C9—H9	118.00
O2—C5—C4	114.42 (12)	C10—C11—H11	119.00
O2—C5—C6	125.10 (12)	C12—C11—H11	119.00
C4—C5—C6	120.47 (13)	C11—C12—H12	120.00
C1—C6—C5	120.36 (12)	C13—C12—H12	120.00
N1—C9—C10	124.02 (14)	C13—C14—H14	120.00
C9—C10—C11	119.63 (13)	C15—C14—H14	120.00
C9—C10—C15	122.79 (13)	C10—C15—H15	119.00
C11—C10—C15	117.58 (13)	C14—C15—H15	119.00
C10—C11—C12	121.91 (13)	O3—C16—H16A	110.00
C11—C12—C13	119.56 (12)	O3—C16—H16B	110.00
O3—C13—C12	124.27 (12)	C17—C16—H16A	110.00
O3—C13—C14	116.29 (12)	C17—C16—H16B	110.00
C12—C13—C14	119.44 (13)	H16A—C16—H16B	108.00
C13—C14—C15	120.28 (12)	N2—C18—H18	127.00
C10—C15—C14	121.14 (12)	C17—C18—H18	127.00
O3—C16—C17	109.84 (12)	N2—C19—H19A	109.00
N4—C17—C16	123.06 (13)	N2—C19—H19B	109.00
N4—C17—C18	108.21 (13)	C20—C19—H19A	109.00
C16—C17—C18	128.49 (13)	C20—C19—H19B	109.00
N2—C18—C17	105.40 (12)	H19A—C19—H19B	108.00
N2—C19—C20	112.12 (14)	C19—C20—H20	118.00
C19—C20—C21	123.90 (17)	C21—C20—H20	118.00
C1—C2—H2	120.00	C20—C21—H21A	120.00
C3—C2—H2	120.00	C20—C21—H21B	120.00
C2—C3—H3	120.00	H21A—C21—H21B	120.00
			
C7—O1—C4—C5	173.80 (13)	C2—C3—C4—C5	−0.9 (2)
C7—O1—C4—C3	−4.8 (2)	O1—C4—C5—C6	−178.56 (12)
C8—O2—C5—C6	2.3 (2)	O1—C4—C5—O2	0.26 (17)
C8—O2—C5—C4	−176.48 (13)	C3—C4—C5—O2	178.94 (13)
C16—O3—C13—C12	12.0 (2)	C3—C4—C5—C6	0.1 (2)
C16—O3—C13—C14	−169.30 (13)	C4—C5—C6—C1	0.3 (2)
C13—O3—C16—C17	176.09 (12)	O2—C5—C6—C1	−178.40 (12)
C1—N1—C9—C10	−176.54 (14)	N1—C9—C10—C15	20.6 (2)
C9—N1—C1—C2	−139.67 (14)	N1—C9—C10—C11	−160.07 (15)
C9—N1—C1—C6	43.7 (2)	C9—C10—C15—C14	−179.39 (14)
C18—N2—N3—N4	−0.57 (17)	C9—C10—C11—C12	177.46 (14)
N3—N2—C19—C20	50.9 (2)	C11—C10—C15—C14	1.3 (2)
C18—N2—C19—C20	−133.53 (16)	C15—C10—C11—C12	−3.2 (2)
C19—N2—N3—N4	175.73 (14)	C10—C11—C12—C13	2.5 (2)
C19—N2—C18—C17	−175.21 (14)	C11—C12—C13—C14	0.2 (2)
N3—N2—C18—C17	0.71 (17)	C11—C12—C13—O3	178.86 (14)
N2—N3—N4—C17	0.19 (17)	C12—C13—C14—C15	−2.1 (2)
N3—N4—C17—C18	0.25 (18)	O3—C13—C14—C15	179.18 (13)
N3—N4—C17—C16	−174.51 (15)	C13—C14—C15—C10	1.3 (2)
C6—C1—C2—C3	−0.9 (2)	O3—C16—C17—N4	−51.4 (2)
C2—C1—C6—C5	0.1 (2)	O3—C16—C17—C18	134.98 (16)
N1—C1—C2—C3	−177.66 (13)	N4—C17—C18—N2	−0.58 (17)
N1—C1—C6—C5	176.80 (12)	C16—C17—C18—N2	173.81 (15)
C1—C2—C3—C4	1.3 (2)	N2—C19—C20—C21	−137.14 (18)
C2—C3—C4—O1	177.60 (14)		

### Hydrogen-bond geometry (Å, º)

Cg2 is the centroid of the C1–C6 benzene ring.

**Table:**

*D*—H···*A*	*D*—H	H···*A*	*D*···*A*	*D*—H···*A*
C19—H19*A*···O2^i^	0.97	2.54	3.385 (2)	146
C16—H16*A*···*Cg*2^ii^	0.97	2.87	3.6647 (18)	140

Symmetry codes: (i) *x*+1, *y*−1, *z*+1; (ii) −*x*+1, −*y*+1, −*z*+1.
